# Therapeutic efficacy of omalizumab in children with moderate-to-severe allergic asthma combined with chronic sinusitis

**DOI:** 10.3389/falgy.2023.1236798

**Published:** 2023-10-16

**Authors:** Weikun Chong, Hailang Li, Juan Wang

**Affiliations:** Department of Pediatrics, BenQ Medical Center, The Affiliated BenQ Hospital of Nanjing Medical University, Nanjing, China

**Keywords:** omalizumab, Chinese children, allergic asthma, chronic sinusitis, lung function

## Abstract

**Background:**

Omalizumab has been approved for treating moderate-to-severe asthma in children aged over 6 years. Its application to asthmatic children with other allergic diseases has been rarely explored. The present study aims to explore the therapeutic efficacy of omalizumab in children with moderate-to-severe allergic asthma combined with chronic sinusitis.

**Methods:**

The clinical data of children diagnosed with moderate-to-severe allergic asthma combined with chronic sinusitis and treated with omalizumab between September 2020 and April 2022 were retrospectively analyzed. Lung function indexes such as Childhood Asthma Control Test (C-ACT) scores, fractional exhaled nitric oxide (FeNO), and forced expiratory volume in the first second (FEV_1_) percent predicted (FEV_1_%pred), small airway function indexes, and the clinical symptoms of chronic sinusitis were analyzed.

**Results:**

A total of 26 children were observed for 16 weeks. After 16 weeks of omalizumab treatment, the significantly increased C-ACT scores (15.57 ± 3.25 points vs. 24.98 ± 5.21 points, *F* = 15.7112, *P *< 0.001) and decreased FeNO (31.55 ± 15.57 ppb vs. 19.86 ± 9.80 ppb, *F* = 4.4265, *P *= 0.0022), compared with those at baseline, were suggestive of well-controlled symptoms of asthma and improved lung function. FEV_1_%pred and FEV_1_/forced vital capacity (FVC) ratio (the ratio of the forced expiratory volume in the first 1 s to the forced vital capacity) increased after omalizumab treatment, although no significant differences were detected (*P *= 0.9954 and 0.9382, respectively). Peak expiratory flow (PEF) percent predicted (PEF%pred) and forced expiratory flow at 75% of FVC (FEF_75%_), 50% of FVC (FEF_50%_), and 25%–75% of FVC (FEF_25%–75%_) significantly increased after omalizumab treatment (*P *= 0.0477, <0.001, <0.001, and <0.001, respectively). Visual analog scale scores significantly decreased after omalizumab treatment (6.40 ± 2.98 points vs. 0.85 ± 0.40 points, *t* = 27.2419, *P *< 0.001), suggesting alleviation in the clinical symptoms of chronic sinusitis.

**Conclusion:**

In this study, it was found that omalizumab can effectively alleviate clinical symptoms and improve lung function and quality of life in children with moderate-to-severe allergic asthma combined with chronic sinusitis.

## Introduction

Allergic asthma, a type of asthma caused and/or triggered by inhaled allergens, is characterized by recurrent wheezing, coughing, shortness of breath, and chest tightness. Allergic asthma is the most common type of childhood asthma, with an increasing prevalence in recent years (1, 2). Eosinophilia, mast cell activation, and immunoglobulin E (IgE) elevations caused by allergen exposures are important events during the onset of allergic asthma (3). Childhood asthma often accompanies other allergic diseases such as allergic rhinitis, sinusitis, eczema, atopic dermatitis, and chronic urticaria, which seriously affects the quality of life of children and their parents and brings challenges to clinical management.

Omalizumab is the first targeted drug for the treatment of allergic asthma. It is a recombinant humanized monoclonal antibody that selectively binds to IgE to inhibit its combination with high-affinity receptors on the surface of effector cells, thus preventing effector cell degranulation, inflammatory factor release, and inflammatory cell recruitment (4). The therapeutic efficacy of omalizumab on childhood asthma has been extensively validated (3, 5), although its application in the treatment of children with asthma, combined with other allergic diseases, has been rarely explored. In the present study, we retrospectively analyzed the efficacy of omalizumab in children with moderate-to-severe allergic asthma, combined with chronic sinusitis, thus providing clinical experiences for clinical management.

## Methods

### Study subjects

Children diagnosed with moderate-to-severe allergic asthma and chronic sinusitis were retrospectively recruited in the study at the Allergic Disease Clinic, Department of Pediatrics, BenQ Medical Center, Nanjing. They were treated with omalizumab between September 2020 and April 2022. The inclusion criteria included (i) children aged ≥6 and <18 years; (ii) diagnosis of allergic asthma in accordance with the *Guidelines for the Diagnosis and Treatment of Bronchial Asthma in Children (2016 Edition)* (6), where asthma well controlled by step 3 therapy was defined as moderate (2, 6) and that well controlled (or still uncontrolled) by step 4–5 therapy was defined as severe (6). Chronic sinusitis was diagnosed on the basis of symptoms and signs, combined with the results of nasal endoscopy and computed tomography (CT) scans (7, 8). All recruited children showed no nasal polyps; (iii) a positive allergy test result for serum IgE, a positive skin prick test result, or a positive blood test result for allergen-specific IgE (sIgE) (2); and (iv) children or their guardians were able to cooperate with the questionnaire survey. The exclusion criteria included children (i) exhibiting allergy to the active ingredients of omalizumab or any other excipients; (ii) who had complicated conditions such as chronic lung disease, pneumonia, pneumothorax, thoracic deformity, heart failure, and other diseases that may affect cardiopulmonary function; and (iii) who had received a course of omalizumab treatment lasting for less than 4 months. Written informed consent was obtained from the guardians or parents of the recruited children before providing the first omalizumab treatment.

### Study design

The dosage and frequency of omalizumab injections were determined on the basis of instructions for the use of omalizumab, body mass index (BMI), and baseline serum IgE. In general, omalizumab was subcutaneously injected every 2–4 weeks at 150–600 mg per administration. Information on Childhood Asthma Control Test (C-ACT) scores, fractional exhaled nitric oxide (FeNO), lung function indexes, and visual analog scale (VAS) scores for chronic sinusitis was collected before administering omalizumab injections in the 1st, 4th, 8th, 12th, and 16th weeks. Incidentally, all these evaluations are part of our routine practice during follow-up.

### Efficacy evaluation of allergic asthma

The therapeutic efficacy of omalizumab on allergic asthma in children aged 6–11 years and those older than 12 years was assessed using the C-ACT and ACT, respectively. In addition, FeNO and lung function indexes such as forced expiratory volume in the first second (FEV_1_) percent predicted (FEV_1_%pred), forced expiratory volume in the first second (FEV_1_)/forced vital capacity (FVC), and small airway indexes were measured.

### Evaluation of chronic sinusitis

The subjective symptoms of chronic sinusitis in children, such as nasal congestion, runny nose, cough, and headache, were assessed using a VAS scale of 0–10 points and classified into mild (≤3 points), moderate (3 < VAS scores ≤ 7 points), and severe (>8 points) (7).

### Statistical analyses

Statistical analyses were performed using SPSS 20.0. Measurement data that were normally distributed were expressed as $\bar{x}\pm s$, and differences between and among groups were analyzed by using the paired *t*-test and repeated-measures ANOVA, respectively. A score of *P *< 0.05 was considered statistically significant.

## Results

### Population characteristics

A total of 26 eligible children with moderate-to-severe allergic asthma, combined with chronic sinusitis, were recruited, of which 14 were boys and 12 were girls aged between 6 years and 5 months and between 14 years and 9 months, respectively. The median total serum IgE was 490 (117–1,389) IU ml^−1^. Subcutaneous injections of omalizumab were administered in all recruited children as follows: eight children were administered with a dose of 300 mg every 4 weeks, six with a dose of 150 mg every 4 weeks, five with a dose of 450 mg every 4 weeks, five with a dose of 300 mg every 2 weeks, and two with a dose of 600 mg every 4 weeks. Adverse events were not reported during the treatment period. A CT scans of the sinuses were performed in 12 children as follows: five children were scanned for paranasal sinusitis of four-paired sinuses; three for maxillary sinusitis, ethmoid sinusitis, and sphenoid sinusitis; three for maxillary sinusitis and ethmoid sinusitis; and one for maxillary sinusitis. All children had moderate-to-severe asthma, with a course ranging from 6 months to 3 years. Routine treatment of asthma, such as the use of inhaled and intranasal corticosteroids, nasal irrigation, and mucolytics, was provided before administering the omalizumab injections. However, the clinical symptoms of asthma and sinusitis still recurred or persisted.

### Therapeutic efficacy of omalizumab on allergic asthma in children

This study enrolled two children older than 11 years. After the omalizumab injections were administered, their ACT scores demonstrated a significant improvement. In one child aged 12 years and 2 months, the ACT score increased from 18 to 24 points, while in a 14-year and 9-month-old child, the score increased from 21 to 25 points. The C-ACT scores of the remaining 24 children younger than 11 years significantly improved after 16 weeks of omalizumab treatment compared with the baseline (*P *< 0.05). FeNO in all recruited children significantly reduced after omalizumab injections (*P *< 0.05). Lung function indexes, such as FEV_1_%pred, FEV_1_/FVC, Peak expiratory flow (PEF) percent predicted (PEF%pred), forced expiratory flow at 75% of FVC (FEF_75%_), 50% of FVC (FEF_50%_), and 25%–75% of FVC (FEF_25%–75%_), all showed favorable changes after administering the injections, although significant differences were detected only in the last three indexes mentioned (*P *< 0.05, Table 1). Inhaled corticosteroids (ICSs) were withdrawn in 10 children after 12 weeks of omalizumab treatment, and they were withdrawn in the remaining 16 children after 16 weeks of treatment.

**Table 1:** C-ACT scores, FeNO, and lung function indexes before and after omalizumab treatment (*n* = 26).

Time point	C-ACT (points)^a^	FeNO (ppb)	FVC (L)	FEV_1_%pred (%)	FEV_1_/FVC (%)	PEF%pred (%)	FEF_75%_ (%)	FEF_50%_ (%)	FEF_25%−75%_ (%)
Before treatment	15.57 ± 3.25	31.55 ± 15.57	2.06 ± 0.81	90.38 ± 48.87	83.03 ± 21.59	83.60 ± 10.56	69.34 ± 9.08	58.79 ± 7.45	60.05 ± 8.47
4 weeks of treatment	19.66 ± 4.1	27.14 ± 13.39	2.14 ± 0.88	91.24 ± 49.33	83.21 ± 21.63	84.63 ± 10.69	70.13 ± 9.18	59.65 ± 7.56	61.10 ± 8.92
8 weeks of treatment	20.66 ± 4.31	22.17 ± 10.94	2.11 ± 0.93	91.60 ± 49.53	84.56 ± 21.98	86.01 ± 10.86	72.53 ± 9.50	60.41 ± 7.66	62.63 ± 9.25
12 weeks of treatment	22.79 ± 4.75	20.33 ± 10.03	2.17 ± 0.84	93.99 ± 50.82	86.28 ± 22.43	87.94 ± 11.10	74.69 ± 9.78	64.83 ± 8.22	64.07 ± 8.76
16 weeks of treatment	24.98 ± 5.21	19.86 ± 9.80	2.18 ± 0.79	95.66 ± 51.72	87.47 ± 22.74	92.19 ± 11.64	80.21 ± 10.50	69.26 ± 8.78	74.86 ± 10.85
*F*-value	15.7112	4.4265	0.164	0.0495	0.1995	2.4741	5.3374	7.9523	10.727
*P*-value	0.0000	0.0022	0.896	0.9954	0.9382	0.0477	0.0005	0.0000	0.0000

^a^ C-ACT scores were graded in 24 children younger than 11 years.

### Therapeutic efficacy of omalizumab on chronic sinusitis in children

**Figure 1:**
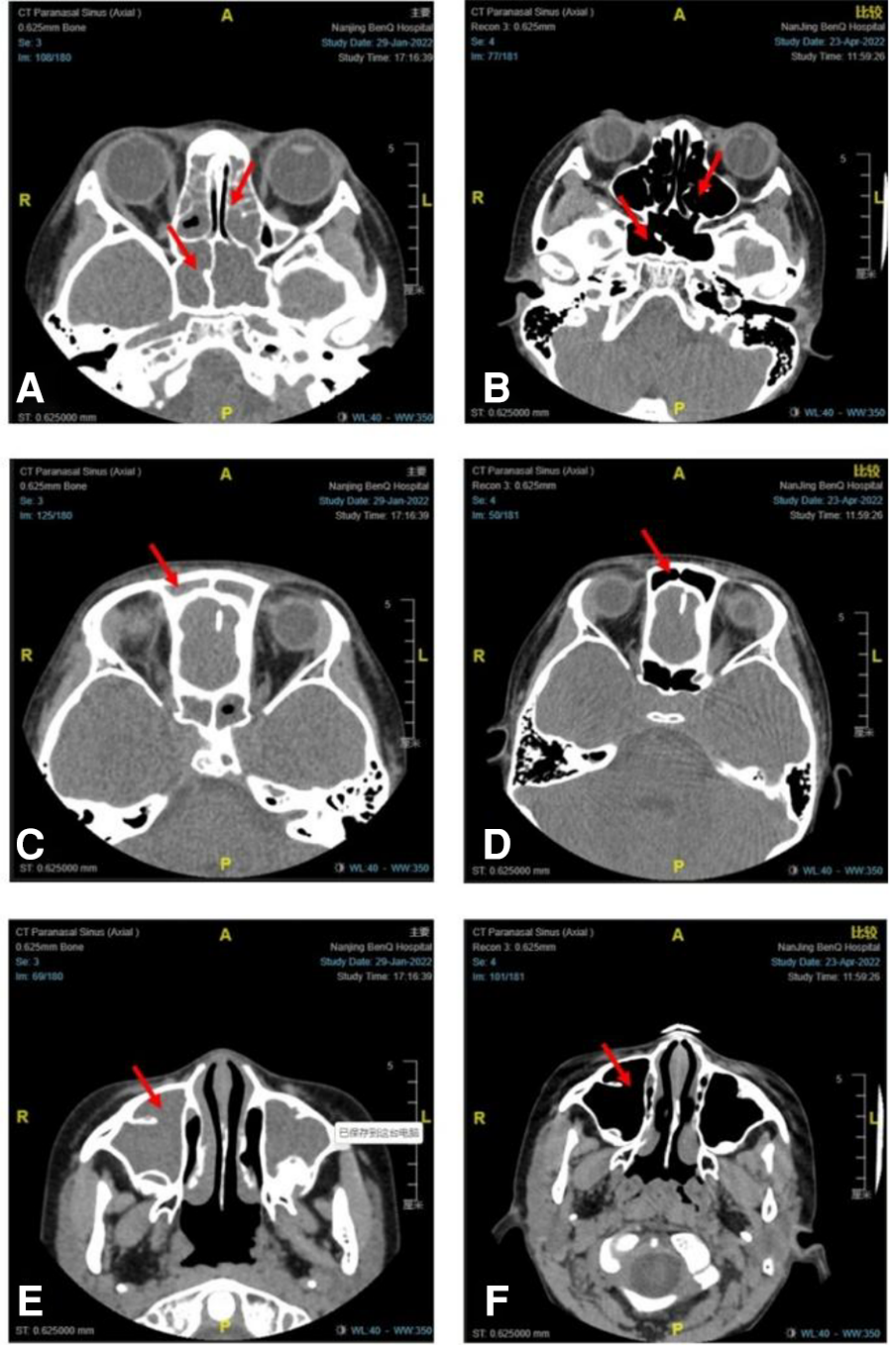
Representative CT scans of sinuses in a 9-year-old boy with moderate-to-severe allergic asthma combined with chronic sinusitis before and after 16 weeks of omalizumab treatment. CT scans of the sphenoid sinus and ethmoid sinus before (**A**) and after (**B**) omalizumab treatment. CT scans of the frontal sinus before (**C**) and after (**D**) omalizumab treatment. CT scans of the maxillary sinus before (**E**) and after (**F**) omalizumab treatment. Red arrows indicate sinusitis.

After 16 weeks of omalizumab treatment, the clinical symptoms of sinusitis in children significantly improved. Withdrawal of nasal corticosteroids was achieved in 12, nine, and five children after omalizumab treatment for 8, 12, and 16 weeks, respectively. The VAS scores of the clinical symptoms of chronic sinusitis significantly reduced after omalizumab treatment in all recruited children (*P* < 0.05, Table 2). A CT scan re-examination of the sinuses was performed in seven children after 16 weeks, all indicating a significant alleviation in chronic sinusitis. Figure 1 shows representative CT scans of sinuses in a 9-year-old boy.

**Table 2:** VAS scores for assessing the clinical symptoms of chronic sinusitis before and after omalizumab treatment (*n* = 26).

Time point	VAS scores (points)
Before treatment	6.40 ± 2.98
4 weeks of treatment	6.11 ± 2.85
8 weeks of treatment	5.66 ± 2.63
12 weeks of treatment	3.34 ± 1.56
16 weeks of treatment	0.85 ± 0.40
*F*-value	27.2419
*P*-value	0.0000

### Adverse events of omalizumab

Local and systemic adverse events were not reported during the 16-week course of omalizumab treatment. Liver and renal dysfunctions were not detected during the regular follow-up testing.

## Discussion

Childhood asthma is mainly controlled by medications of ICSs, ICSs combined with long-acting *β*2 agonists (ICSs-LABAs), and leukotriene receptor antagonists (LTRAs). However, it has been found that the symptoms in some asthmatic children have not been well controlled by these drugs. A multicenter study has reported that asthma is controlled in 19.9% of children aged 2–16 years (9). Children with asthma usually suffer from many comorbidities such as allergic rhinitis, sinusitis, atopic dermatitis, food allergy, obesity, and gastroesophageal reflux disease, which potentially influence the clinical management of asthma and thus pose greater challenges in controlling asthmatic symptoms (10).

Chronic sinusitis and asthma are common heterogeneous diseases in children sharing the same pathogenesis. Sinusitis is an inflammation of the nasal cavity and sinus mucosa. Asthma is a chronic inflammatory disease of the lower respiratory tract. Closely linked to allergen exposures, both sinusitis and asthma often coexist and interrelate with each other (11). At present, the causal relationship between sinusitis and asthma is controversial. However, previous findings have validated their epidemiological correlation (12, 13). Approximately 40% of adults and children with asthma suffer from the comorbidity of sinusitis, and the incidence rate of sinusitis is even higher in patients with severe asthma (14). For children and adolescents with poorly controlled asthma, nasal endoscopy is recommended to identify occult or obvious sinusitis, and CT examinations are performed if necessary (15).

However, the clinical symptoms of upper airway inflammation in children with allergic asthma are usually poorly controlled because of the lack of clinical experience in the management of chronic sinusitis. Intranasal corticosteroids and nasal irrigation with topical antibiotics or normal saline are common therapeutic strategies for chronic sinusitis in children (16). Nevertheless, a single treatment usually cannot reap an acceptable therapeutic outcome because of the complicated pathogenesis of sinusitis and persistent inflammatory response.

Precision medicine and immunotherapy have transformed the treatment of airway inflammatory diseases (17). Omalizumab, a recombinant humanized anti-IgE monoclonal antibody, is the first targeted drug for the treatment of asthma. After 10 years of its clinical application, omalizumab has been validated for its efficacy in controlling the symptoms of asthma, preventing the aggravation of asthma, and lowering the medical utilization and the rate of class absence (18). Previous studies have demonstrated that omalizumab treatment alleviates not only asthma symptoms but also the outcomes of asthma comorbidities (14, 19). Similar to the immune process of asthma and allergic rhinitis, IgE is of great significance in the Th2 responses of chronic sinusitis. Meanwhile, serum IgE level is correlated with the inflammation severity of the mucous membrane lining the sinuses. Considering the pathogenic similarities shared by asthma and chronic sinusitis, omalizumab injections also provide clinical benefits to patients with chronic sinusitis (20). In December 2020, the Food and Drug Administration (FDA) approved omalizumab for the treatment of chronic rhinosinusitis with nasal polyps (CRSwNP) (21). All recruited asthma children had complications of chronic sinusitis. Their nasal symptoms of sinusitis were recurrent with a long-period course ranging from 6 months to 3 years, even after ICSs and nasal irrigation were used, which significantly influenced the quality of life. After omalizumab treatment, significantly decreased VAS scores were indicative of relieved symptoms of sinusitis. A total of 12 children achieved withdrawal of intranasal corticosteroids after 8 weeks of omalizumab treatment. The sinuses of seven children were re-scanned using CT after 16 weeks of treatment, suggesting an obvious relief in inflammation in the sinuses. However, we were unable to provide the Lund–Mackay score to quantitatively reflect the change in chronic sinusitis before and after treatment. At 16 weeks of omalizumab treatment, all recruited children withdrew intranasal corticosteroids. So far, they have been regularly followed up in our pediatric outpatient clinic, and they have been presenting stable symptoms of allergic asthma and chronic sinusitis. Some children were also given intranasal corticosteroids during allergy seasons, and their symptoms could be well controlled within 1 week. Sui et al. (22) consistently reported the acceptable efficacy and safety of omalizumab in the treatment of asthma combined with refractory sinusitis in Chinese patients. An anti-IgE therapy is recommended for children with allergic asthma combined with chronic sinusitis because they are generally poorly responsive to conventional treatment.

Several multicenter, randomized, double-blinded controlled studies have shown that ICSs, combined with omalizumab, can effectively control the clinical symptoms of moderate-to-severe allergic asthma in children and reduce the incidence of an acute attack of asthma (23, 24). In the present study, significantly increased C-ACT and ACT scores after omalizumab injections suggested that omalizumab treatment effectively relieved asthma symptoms and improved the quality of life of affected children. The role of omalizumab in enhancing the lung function of asthma children has been found to be controversial. Oliveira et al. (25) reported that omalizumab treatment increases FEV_1_ in obese patients with severe asthma. Licari et al. (26) revealed that FEV_1_%pred in children with severe allergic asthma increases from 79% to 91% after 12 months of omalizumab treatment. However, a Japanese research team demonstrated that omalizumab treatment did not significantly improve FEV_1_%pred and FEF_25%–75%_pred in asthmatic children (27). In the present study, both FEV_1_%pred and FEV_1_/FVC increased after 16 weeks of omalizumab treatment, although no significant differences were detected. PEF%pred, FEF_75%_, FEF_50%_, and FEF_25%–75%_ in children significantly increased after omalizumab treatment, suggesting that omalizumab mainly improved the small airway function. In addition, the significantly decreased FeNO after omalizumab treatment could be attributed to the removal of eosinophilic airway inflammation.

Taken together, omalizumab injections effectively relieved the symptoms of asthma and sinusitis and improved lung function and quality of life in children with moderate-to-severe allergic asthma, combined with chronic sinusitis, with no local and systemic adverse events. Notably, this study was limited by a small sample size, a short follow-up period, and a lack of the Lund–Kennedy score for objectively evaluating endoscopic mucosal morphology of sinusitis. The small sample size and the selection of patients with only moderate asthma may limit our results about the impact of omalizumab on some clinical parameters such as lung function FEV1%pred and FEV1/FVC. Therefore, our findings should be further validated in a large-scale study using a rigorous study design.

## Data Availability

The original contributions presented in the study are included in the article/Supplementary Material, further inquiries can be directed to the corresponding author.
